# I’m Not Good for Anything and That’s Why I’m Stressed: Analysis of the Effect of Self-Efficacy and Emotional Intelligence on Student Stress Using SEM and QCA

**DOI:** 10.3389/fpsyg.2020.00295

**Published:** 2020-03-13

**Authors:** Diego Navarro-Mateu, Lucía Alonso-Larza, María Teresa Gómez-Domínguez, Vicente Prado-Gascó, Selene Valero-Moreno

**Affiliations:** ^1^Department of Educational Psychology and Special Educational Needs, Faculty of Psychology, Teaching and Educational Sciences, Catholic University of Valencia, Valencia, Spain; ^2^Department of Social Psychology, Faculty of Psychology, University of Valencia, Valencia, Spain; ^3^Department of Personality, Assessment and Psychological Treatments, Faculty of Psychology, University of Valencia, Valencia, Spain

**Keywords:** stress, self-efficacy, emotional intelligence, qualitative comparative analysis, structural equation models

## Abstract

Stress negatively affects the well-being and the quality of life of the society. Specifically in the academic context, it is relevant to analyze its levels due to its impact on performance and learning. There are factors that affect the said stress including, among others, self-efficacy, and emotional intelligence. The purpose of this study is to analyze how emotional intelligence and perceived self-efficacy affect student stress. In order to show this influence, two complementary methodologies are implemented: the structural equation models (SEMs) and the comparative qualitative analysis (QCA). A total of 477 students (85% of women) from a private University of Valencia participated in the study, with ages ranging from 18 to 53 years old (*M* = 21.57, *SD* = 3.68). The assessment instruments used were as follows: Emotional Intelligence Scale (TMMS-24) to measure emotional intelligence; General Self-Efficacy Scale (GSS) to measure self-efficacy; and Perceived Stress Scale (PSS) to measure stress. The results in the SEM endorse the hypotheses that emotional clarity and self-efficacy are negatively related to stress and positively related to emotional attention (EA), explaining 25% of the variance. The QCA results show that none of the variables is a necessary condition for inducing stress. Nevertheless, different combinations of these variables are sufficient conditions to explain 35% of the high stress levels. The most important combination over high stress levels seems to be the interaction between high levels of EA and low levels of self-efficacy. Regarding the low levels of perceived stress, there are sufficient conditions to explain 50% of them. Mainly, the most important interaction is between low levels of self-efficacy and low levels of EA. The comparison of both methodologies enables the broadening of new horizons at the methodological level applicable to different contexts.

## Introduction

Stress is one of the most studied psychosocial factors related to many aspects of life, such as work development (or job performance) (Burton et al., 2017), academic performance (Caballero et al., 2015), health and emotional well-being (Dhabhar, 2014; Klein et al., 2016; Reed et al., 2016), illness (Cohen et al., 2007), or adaptation to specific situations (Boswell et al., 2013; Banerjee and Chatterjee, 2016; Stuart et al., 2016).

In general, stress refers to the manner in which an individual responds to certain environmental situations that overwhelm her and that she considers threatening (or intimidating), feeling that her well-being is compromised. Therefore, it is not a uniform process, but rather an experience depending on the interaction of different factors. Among these factors, there are environmental conditions and individual traits, such as attitudes, motivations, emotional responses, and, specifically, the manner of dealing with situations (El-Ghoroury et al., 2012; Mahmoud et al., 2012; Boswell et al., 2013; Folkman, 2013; Gómez et al., 2014).

The university context is a potentially stressful environment, in which the student may experience a certain lack of control in light of the demands and requirements of the environment (Pulido Rull et al., 2011; De la Fuente, 2015). According to several studies, stress in the academic context is closely related to overload of academic tasks, time constraints (Arribas, 2013; Alfonso et al., 2015), high frequency of evaluations, work and daily tasks pressure, competitiveness, and other aspects that may be perceived by the subject as obstacles that exceed her abilities to achieve success (Fernández et al., 2015). Previous research show that academic stress may be present in the student at any stage of her academic life (Putwain, 2007), confirming its increase as the subject progresses academically and reaches higher levels, such as the university level, where the workload and responsibility, together with the changes that the student experiences in her life, generate an increase in academic stress (Berrio and Mazo, 2012).

Any stimulus or situation faced by an individual has the potential to become a stressful event, capable of producing in the individual a decrease in her physical and mental health, well-being, or quality of life (Lazarus, 2000). For a stimulus or situation to be valued as such, this depends on the transactions or exchange processes that take place between the individual and the context (Lazarus and Folkman, 1986; Lazarus, 2000). In this regard, the transactional stress model (Lazarus and Folkman, 1986) states that evaluative and attribution processes are essential in stress perception (Lazarus and Folkam, 1984). This model considers two sequential appraisal processes: in the first place, the so-called primary appraisal, where the significance of the stressor or the stressful event is evaluated, and in the second place, the so-called secondary appraisal, where the grade of control over the stressor and the available resources are evaluated. In this sense, in the presence of a stimulus or stressful situation, the individual will judge it as relevant if it produces a significant change in her relationship with the environment or if it alters her state of balance (well-being). If the stimulus is judged as relevant, then the individual will proceed to analyze the degree of controllability she has over that situation. She will also analyze the available options and resources: those necessary and those available in the individual’s repertoire. After this second appraisal, which, in turn, can modify the primary one, if the stressful stimulus continues to be judged as threatening, the individual will implement a series of coping strategies, depending on the resources available (personal, social, or cultural) and on the degree of controllability of the situation. This will give way to the so-called problem-oriented strategies and strategies oriented toward emotional regulation. According to the success of these strategies, a series of positive or negative coping results will be produced. If the results of coping are negative, there will be an increase in perceived stress.

There are numerous factors that can affect these appraisal processes. Alfonso et al. (2015) point out that, among the factors involved in the stress perceived by students, it is necessary to consider, on the one hand, biological variables, such as age and gender, and socioeconomic variables, such as place of residence or the receipt of financial aid and scholarships; and on the other, psychosocial variables, such as social support, emotional intelligence (EI), self-concept, self-efficacy, coping mechanisms, and psychoeducational strategies, just to name a few examples that must likewise be considered.

Currently, there is evidence regarding the role of personal resources in light of the emergence of stress and the protection of the subject when facing the said stress (Bruehlman-Senecal et al., 2016; Cabanach et al., 2017). Several researches emphasize the influence of personal variables when it comes to cushioning the negative effects of stress in academic contexts (Salanova et al., 2005; Loureiro, 2008). Noteworthy, among others, are the beliefs of self-efficacy (León-Rubio et al., 2011), the coping strategies used by the subject when facing stress (Bonanno and Burton, 2013; Cabanach et al., 2013), or the EI (Aguilar et al., 2014; Bryant and Malone, 2015; Hodzic et al., 2016).

The individual’s EI and her perception of her own abilities (self-efficacy) stand out among the resources that she can use to deal with stressful situations (León-Rubio et al., 2011). For Lazarus and Folkman (1986), in a situation of stress, the assessment that the subject makes about the stressors is very important, and also the set of emotions and feelings that are associated with it. In this regard, an individual’s ability to perceive her own emotions and those of others, and to use them appropriately (key aspects of EI), will influence the subject’s perception of the situation and, therefore, her capacity to face this stress (Min, 2014; Yamani et al., 2014; Extremera et al., 2016). In the same vein, Lazarus and Folkman (1986) highlight the efforts that the individual undertakes at the behavioral and cognitive level to face the stressful situation, as well as the capacity perceived by herself to successfully deal with the environmental conditions, as is the case of the perception of self-efficacy (Martínez et al., 2010; Cabanach et al., 2013).

Insofar as the EI is concerned, the literature suggests that high EI levels are related to a lower perception of stress (Extremera et al., 2016; Serrano and Andreu, 2016; Zhang et al., 2016) as well as to greater levels of happiness and satisfaction (Ruiz-Aranda et al., 2014).

This positive influence of EI as regards self-perceived stress levels is probably due to the relationship between the EI and the coping strategies used. In general, high levels of EI are related to the use of more adaptive strategies (Augusto-Landa et al., 2011; Fernández-Berrocal et al., 2012), as is the case of coping strategies geared toward contemplation and problem solving. On the contrary, low levels of EI would be associated with strategies based on avoidance, superstition, and/or rumination (Martínez et al., 2010).

One of the most important models of EI is the Mayer and Salovey model (1990) (Salovey et al., 1995), which is included within the models of ability. EI is understood as the ability to perceive and express emotions; the ability to use these emotions to facilitate thinking comprehension as well as the motive for the emotion; and the ability to regulate both one’s own emotions and those from others. Thus, this model emphasizes the adaptive use of emotions, which are understood as a facilitator for a more effective reasoning. Likewise, it is encompassed from this model that the EI would form the following dimensions: emotional attention (EA), which is the ability to cater and to observe one’s own emotions and feelings; emotional clarity (EC), which refers to the understanding of one’s own emotional states; and emotional reparation, which refers to the person’s beliefs about her own ability to regulate feelings and emotions. While both higher levels of EC and emotional reparation suggest higher levels of EI, this is not the case for EA. For this case, high and low levels of EA would indicate inadequate levels of EI, since excessive EA can lead to the so-called rumination. For this reason, these three dimensions should all be considered in relation with EI separately (Guerra-Bustamante et al., 2019; Martínez-Marín and Martínez, 2019). According to this, the literature suggests (Augusto-Landa et al., 2011; Serrano and Andreu, 2016) that while the EA dimension maintains a positive relationship with perceived stress (Davis and Nichols, 2016; Villanueva et al., 2017), the dimensions of EC and emotional repair (ER) show a negative relationship with the mentioned perceived stress (Aguilar et al., 2014; Ruiz-Aranda et al., 2014; Bryant and Malone, 2015; Perera and DiGiacomo, 2015; Schönfeld et al., 2016; Zeidner and Matthews, 2016). Low scores in the different components of EI are related to high levels of perceived stress (Austin et al., 2010), but in the case of EA, this relation is more complex. Even when EA was classified in three levels: low, adequate, and excessive attention to emotions (inverted U-effects), no significant association between the levels of EA and perceived stress was found (Guerra-Bustamante et al., 2019).

All this seems to be observed in different areas, but especially in the academic context (Ruiz-Aranda et al., 2014; Perera and DiGiacomo, 2015; Serrano and Andreu, 2016; Zhang et al., 2016). In the said context, it would appear that, in relation to the perception of stress, the ability to manage one’s own emotions, such as the attention that the individual lends to her feelings and comprehension thereof, is very influential (Augusto-Landa et al., 2011).

Similarly, the literature highlights the close relationship between beliefs of self-efficacy and the stress perceived by the subject in different areas (Bresó et al., 2011; Hahn et al., 2011). The evidence suggests that high levels of self-efficacy are associated with low levels of perceived stress (Karademas and Kalantzi-Azizi, 2004; Zhao et al., 2015; Dominguez-Lara, 2018). In consonance with this, low levels of efficacy expectations are related with high levels of anxiety and stress (Soysa and Wilcomb, 2015; Valle et al., 2015; Grøtan et al., 2019). The reason for these associations is the role of expectations as protective or buffering factors against stress (Frick et al., 2016). Thus, several studies (Paoloni and Bonetto, 2013; Cabanach et al., 2017) state that beliefs related to perceptions of competence influence the cognitive assessment that the subject makes as regard the demands of the environment, as well as the activation of certain strategies depending thereon. Specifically, it seems that people with high levels of perceived efficacy show greater confidence when responding to the demands of their environment, which, in turn, influences the perception of a threat (Cabanach et al., 2017). Evidence indicates that subjects with high self-efficacy beliefs interpret context demands as less stressful and threatening than subjects with low self-efficacy beliefs (Aguilar et al., 2014; Nastaskin and Fiocco, 2015; Zhao et al., 2015; Schönfeld et al., 2016).

This situation is observed and has been widely perceived in the university context (Aguilar et al., 2014; Cabanach et al., 2017), confirming that the levels of self-efficacy beliefs correlate negatively and significantly with the levels of self-perceived stress (Zhao et al., 2015; Schönfeld et al., 2016). It would seem that subjects with high self-efficacy beliefs interpret the demands of the context as less stressful and threatening (or intimidating) than those who show low self-efficacy beliefs (Aguilar et al., 2014; Zhao et al., 2015; Schönfeld et al., 2016) and more frequently use optimistic and problem-solving strategies. Several researches try to verify the possible relationship between both variables EI and self-efficacy beliefs (Aguilar et al., 2014; Joseph et al., 2015; Honicke and Broadbent, 2016). In general, there seems to be a mutual influence between emotions and self-efficacy beliefs. On the one hand, a high level of self-efficacy beliefs is directly related to a greater experience of positive affection, as well as a significantly lesser experience of negative affection (Sansinenea et al., 2008). On the other hand, positive moods increase the levels of perceived competence, while the experience of negative moods decreases this perception (Checa and Fernández-Berrocal, 2019). All these studies consider that EI plays a very important role in self-efficacy beliefs, because a good handling of emotions increases the feeling of competence and the perception of success toward academic tasks, and also toward tasks of other kind (Khani and Mirzaee, 2015; Morales-Rodríguez and Pérez-Mármol, 2019).

In general, the literature reflects the positive relationship between EI and self-efficacy and the negative relationship of these two variables with stress experience (Aguilar et al., 2014; Serrano and Andreu, 2016; Cabanach et al., 2017). In this fashion, the studies undertaken with university students underline the existence of significant relationships between the perception of emotions, self-efficacy, and stress experience (Martínez et al., 2011; Alfonso et al., 2015).

The Lazarus and Folkman (1986) transactional stress model explains how an individual’s differences, determined by the subject’s emotional involvement and her ability to clarify and regulate her own emotions, may play a key role in how she evaluates the situation as more or less threatening (Augusto-Landa et al., 2011; Ruiz-Aranda et al., 2014; Serrano and Andreu, 2016; Matthews et al., 2017). Similarly, the control over one’s own emotions and the capacity to objectify them play a very important role in the elaboration of self-efficacy beliefs. The individual makes an assessment of the resources at her disposal to face a situation more or less in accordance with reality, as well as an assessment of the possibilities of successfully solving the task using such resources (Cabanach et al., 2017). Therefore, the introduction of the emotion factor can help to explain how the subject values a situation and the extent to which she considers herself capable of facing it successfully (Salguero et al., 2012). In addition, the person–situation interaction model suggests that the nature of stressful situations moderates the activation of coping strategies and styles (Villasana et al., 2016).

**FIGURE 1:**
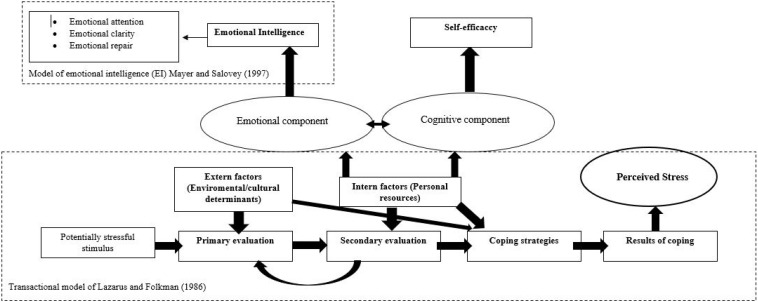
Own model based on the transactional model of stress of Lazarus and Folkman (1986) and emotional intelligence of Mayer and Salovey (1997). Discontinuous lines refer to the fact that it is part of a theoretical model.

In other words, both EI and self-efficacy beliefs would influence students’ cognitive appraisal of the situation, the so-called primary appraisal. Although they could also affect the secondary appraisal, or become a resource to face the situation, self-efficacy would actually play a key role in the so-called secondary appraisal by influencing the individual’s assessment of her coping resources (Figure 1).

Therefore, low levels of EI could provoke a subject’s appraisal of the situation as more threatening, while such levels coupled with a low perception of self-efficacy would influence a perception of a scarcity of coping resources for her. These effects would lead to higher levels of perceived stress. Specifically, the literature suggests that it is expected that EA will be positively and significantly related to perceived stress, since this excess of EA seems to be related to rumination and the use of less functional, or healthy, strategies (LeMoult et al., 2013; Martínez-Marín and Martínez, 2019). It also suggests that EC and ER will be negatively related to stress, since they are resources that facilitate the use of more functional strategies by improving the management of stress (Bryant and Malone, 2015; Perera and DiGiacomo, 2015; Schönfeld et al., 2016; Zeidner and Matthews, 2016) (hypothesis 1). Similarly, as discussed above, given that self-efficacy appears to affect the individual’s self-perceived ability to cope with stress and her assessment of the resources available to her (Soysa and Wilcomb, 2015; Valle et al., 2015; Grøtan et al., 2019), it is expected that self-efficacy will be negatively related to the levels of perceived stress (hypothesis 2).

Despite the importance of stress in the lives of individuals (Moore et al., 2016) and specifically in the case of university students (Lapointe et al., 2018), and the role played by EI as well as self-efficacy in the said stress, few studies have simultaneously analyzed the role played by both aspects as regard stress (Aguilar et al., 2014). In response to this, the present research aims to determine the role played by self-efficacy and EI over perceived stress. In this regard, the Lazarus and Folkman transactional stress model (Lazarus and Folkman, 1986) considers that certain personal and social resources may act as protection from stress or risk factors. For Lazarus and Folkman (1986), in a situation of stress, the assessment that the subject makes of the stressors, as well as the set of emotions and feelings associated with it, is very important. In this regard, the ability of the individual to perceive her own emotions and those of others and to use them appropriately (key aspects of EI) will influence the perception of the situation by the subject and, therefore, in her capacity to face the said stress (Min, 2014; Yamani et al., 2014; Extremera et al., 2016), as well as the perception of self-efficacy (Martínez et al., 2010; Cabanach et al., 2013).

Likewise, most of the existing studies have focused on the so-called linear models, which are based on the observation of linear relationships between the variables under study, obviating other types of non-linear interaction. By contrast, qualitative comparative analysis (QCA) can study beyond the individual contribution of each one of the variables to observe possible non-linear interactions between them (Villanueva et al., 2017; Castellano Rioja et al., 2019; Crespo-Hervás et al., 2019; Giménez-Espert et al., 2019).

### Linear Models Versus QCA

In the field of psychology, most of the research starts from linear models, such as linear regression models and their successive evolutions: structural equation models (SEM) and PLS models (SEM-PLS). Linear models are based on the individual contribution of each variable and do not take into account (*a priori*) the interaction or the combination between the different variables studied. They do not have into consideration the different possible combinations or paths that can lead to the same result (equifinality). Neither they take into account that the variables, or factors, that explain a given result may not be the same as those that explain this result in the opposite direction (Ragin, 2008; Eng and Woodside, 2012; Boquera et al., 2016). Also, in lineal models, there is a limit to the number of interaction effects that can be included in an analysis (Seawright, 2005; Vis, 2012).

By contrast, the QCA is an analytical technique that does allow the in-depth analysis of how a combination of causal conditions (variables) contributes to a given outcome. QCA models are based on Boolean logic and assume the influence of a particular attribute, or attributes, on a specific outcome. They are based on the way these attributes are combined (variables or, according to specific terminology, conditions), rather than on the individual contribution of each variable. This technique also allows to perceive different combinations or paths that can lead to the same result (equifinality). It also makes it possible to analyze that, although some variables may give rise to a given result, it does not necessarily imply that the same variables are equally relevant to obtaining the opposite result when in the opposite direction (Ragin, 2008). In addition, QCA addresses multiple contextual causes in a simple way, allowing for greater horizontal complexity than linear models (Seawright, 2005; Vis, 2012). It offers a more systematic way of analyzing complex causality and logical relationships between causal conditions and variable outcomes than the linear models (Legewie, 2013). In addition, it also allows working with small samples (Ragin, 2008; Eng and Woodside, 2012). The analysis establishes the so-called *necessary conditions*, which are those causes that must always be present in order for a specific result to be given; and the so-called *sufficient conditions*, which can give rise to a given result, although they do not always have to be present for a result to be given. QCA models make it possible to identify the percentage of explained variance (coverage), as well as the indicators of goodness of adjustment (consistency) (Ragin, 2008; Eng and Woodside, 2012). According to this, literature recommends the use of the two methodologies in a complementary manner, despite the differences between linear models and QCA (Seawright, 2005; Vis, 2012; Boquera et al., 2016; Villanueva et al., 2017; Castellano Rioja et al., 2019; Giménez-Espert et al., 2019). SEMs will offer different but complementary results to those provided by the QCA.

As mentioned before, the objective of the present study is to analyze the influence of EI and self-efficacy in the prediction of stress levels in university students, while comparing two complementary methodologies: the SEM and QCA. It came up by the given importance of stress and its prediction in the lives of individuals, and especially in academic contexts; by the impact that EI and self-efficiency can have on such stress; by the scarcity of studies in this context that have addressed the role that both personal factors can have in the level of self-perceived stress; and because of the need to combine different, complementary, methodological approaches. On the basis of this general objective and the theoretical framework considered, as indicated above, two hypotheses have emerged—H1: EA will be positively and significantly related to perceived stress, while EC and ER will be negatively related to stress. H2: Self-efficacy will be negatively related to the levels of perceived stress.

## Materials and Methods

### Participants and Procedure

This study involved 477 university students from the Valencian Community. Specifically, the students belonged to the Faculty of Psychology, Teaching and Educational Sciences of a private University of Valencia. The average age of all the subjects surveyed was 21.57 years old (*SD* = 3.68), with a minimum age of 18 and a maximum age of 53 years. The percentage of men surveyed is 14.16%, while that of women is 85.84%. Due to the nature of the study, participants answered voluntarily and the questionnaires were anonymous. The study was approved by the bioethics committee of the Catholic University of Valencia (PRUCV/2015/660). All participants received detailed information about the aims and procedures and were informed of confidentiality. Data collection and data analysis took place between October 2016 and January 2017.

### Statistical Analysis

First descriptive analyses of the participants were estimated, and then, calibration values for fsQCA were calculated; after that, SEM and a fuzzy-set QCA (fsQCA) were performed. In the SEM, the estimate provided by the robust method of maximum likelihood estimation (ML) recommended to correct the possible absence of multivariate normality was applied in all cases. The adequacy of the model was verified through the significance of chi-square and its robust correction provided by Satorra-Bentler (S-Bχ^2^) (Satorra and Bentler, 1994; Hu and Bentler, 1995). Other coefficients were also calculated to check the suitability of the models proposed, such as the ratio of χ^2^ and its degrees of freedom (χ^2^/df) and S-BX^2^ and its degrees of freedom, being acceptable values lower than 5 (Carmines and McIver, 1981; Brynne, 2009). Finally, the coefficients of the robust goodness-of-fit indices of the proposed models were checked: the comparative fit index (CFI) and the incremental fit fix (IFI). For these indicators, values above 0.90 are considered as indicators of good fit (MacCallum and Austin, 2000; Kline, 2005). Finally, the root mean-square error of approximation (RMSEA) is shown, with scores lower than 0.08 being considered as indicators of good fit (Browne and Cudeck, 1993).

For QCAs, raw data from the participants’ responses were transformed into fuzzy set responses. First, as the literature suggested, all missing data were eliminated and all questionnaire constructs, or scores (variables), were calculated by multiplying the scores of the items (Boquera et al., 2016; Villanueva et al., 2017; Giménez-Espert et al., 2019). Then, the values were recalibrated between 0 and 1 (Ragin, 2008) by means of Claude and Christopher’s fsQCA 2.5 software (2014), taking into consideration the three thresholds that the literature suggests (Woodside, 2013): 10% (low agreement or totally outside the set), 50% (intermediate level of agreement, neither inside nor outside the set), and 90% (high agreement or totally within the set). In consonance with the literature, once the responses were transformed, necessary and sufficient condition tests were performed in order to evaluate the effect of EI and self-efficacy on a particular outcome (high levels of perceived stress) and on the absence of it (low levels of perceived stress). On the one hand, a condition is necessary when it must always be present for the occurrence of a particular outcome (consistency in necessary analysis must be greater than 0.90). On the other hand, a condition (or combination of conditions) is sufficient when it gives rise to a given result, but it can also be reached by other conditions or combinations thereof (Ragin, 2008). The fsQCA generates three possible solutions: complex, parsimonious, and intermediate. The complex solution is the most restrictive, and the parsimonious solution is the less restrictive. Previous studies (Ragin, 2008) recommend to consider the intermediate solution, the one presented here. On sufficiency analysis, the overall solution coverage considers the variance explained by all the paths, or combinations of conditions (variables), while the overall solution consistency considers the possible reliability or fit of a model. When the total consistency of the model is greater than 0.75, a sufficiency outcome is considered adequate. In addition, raw coverage indicates how many cases, or observations, can be explained by each path (condition or combination of conditions), while the unique coverage expresses the number of observations (variance) that can be explained by a particular combination of conditions, but not by another combination of conditions. Finally, the consistency indicates reliability or fit of a path (condition or combination of conditions) that would explain an observation (Eng and Woodside, 2012).

Statistical Package for the Social Sciences (SPSS, Version 23, ©IBM) was used to perform descriptive analyses and calibration values. EQS (Structural Equation Modelling Software, Version 6.3, Bentler, 1985-2016, Multivariate Software Inc.) was used to evaluate the psychometric properties of instruments and structural equations models. Nevertheless, fsQCA (version 2.5, ©Raging and David, 1999–2008; Claude and Christopher, 2014) was used to perform the QCA analysis.

### Measures

#### Trait Meta Mood Scale (TMSS-24) (Salovey et al., 1995)

In this study, the Spanish version of the EI scale (TMMS) from Salovey et al. (1995), adapted by Fernández-Berrocal et al. (2004), was used. It is an instrument composed of 24 items, presented on a 5-point Likert-type scale, where 1 corresponds to strongly disagree and 5 to strongly agree, distributed in three dimensions with eight indicators each. On the one hand, the EA dimension (eight items) refers to the ability to feel and express feelings in an appropriate way. The EC dimension (eight items) refers to the ability to understand one’s own emotional states, and finally, the ER dimension (eight items) corresponds to the ability to correctly regulate emotional states. This instrument has shown adequate psychometric properties in previous studies. The definitive model consists of 20 items distributed in the three dimensions proposed by Fernández-Berrocal et al. (2004). This model has shown adequate validity and reliability indicators in previous studies, which also have been obtained in this study: [χ^2^(df) = 516.33(167); S-Bχ^2^ (df) = 406.07(167); χ^2^/df = 3.09; CFI = 0.93; IFI = 0.93; RMSEA = 0.058 (IC = 0.051–0.065); α = 0.87 for the whole scale; α for the attention dimension = 0.86; for the clarity dimension = 0.89; α for the reparation dimension = 0.86].

#### General Self-Efficacy Scale (Schwarzer and Baessler, 1996)

Sanjuán et al. (2000) have validated the adaptation for the Spanish population of this scale. It is a one-dimensional scale and is made up of 10 items, presented on a Likert-type scale with four alternatives of answer, where 1 corresponds to incorrect and 4 to certain. This scale assesses the stable feeling of personal competence to manage a wide variety of stressful situations. It has shown adequate psychometric properties in a sample of university students (Sanjuán et al., 2000). As regard the psychometric properties in the present study, they seem to be adequate: [χ^2^(df) = 122.51(27); S-Bχ^2^(df) = 95.71(27); Bχ^2^/df = 4.54; IFC = 0.94; IFI = 0.94; RMSEA = 0.077 (IC = 0.061–0.094); α = 0.88].

#### Perceived Stress Scale (PSS) (Cohen et al., 1983)

Consisting of a single dimension, it aims to measure the degree to which people perceive their life situation over the past month as stressful. It is a Likert-type scale with five answer options, where 1 corresponds to never and 5 to always. It has been validated in the Spanish context by Remor (2006) both in its extended version (14 items) and in its reduced version (10 or 4 items) and in Spanish university students (Extremera et al., 2007). The versions (i.e., in the 14, 10, and 4 versions) of the scale show good internal consistency (Pedrero and Arroyo, 2010). Following the recommendation of Pedrero Pérez and Olivar (2010), the scale must be considered as a one-dimensional structure. Thus, several re-specifications of the PSS (deleting items 6 and 9) were made to achieve a factorial solution with a good fit for the sample under study. The adequate validity and reliability indicators justify its use in this study: [χ^2^(df) = 103.31(20); S-Bχ^2^(df) = 85.43(20); χ^2^/df = 5.16; CFI = 0.93; IFI = 0.93; RMSEA = 0.085 (IC = 0.067–0.104); α = 0.84].

## Results

First, the main descriptors and calibration values for the variables under study are presented (Table 1).

**TABLE 1:** Main descriptions and calibration values.

	EA	EC	ER	PS	GSE
*M*	84642.40	67595.37	69765.23	3944192.7	44587.88
*SD*	121517.22	95120.6	96867.5	227475151.9	60117.03
Min.	16	8	12	10	27
Max	1620000	390625	390625	1600000000	262144
***Calibration values***					
P10	3379.2	2381.4	1689.6	978432	2190.4
P50	43200	27648	34560	10616832	19683
P90	200000	200000	200000	80356478.6	112465.76

*M, mean; SD, standard deviation; min, minimum; max, maximum; P10, 10th percentile; P50, 50th percentile; P90, 90th percentile; EA, emotional attention; EC, emotional clarity; ER, emotional repair; PS, perceived stress; GSE, general self-efficacy.*

### Structural Equation Model (SEM)

In order to calculate the SEM, the different items that compose the scales of indicators of latent variables and dimensions were determined. As the authors of the scale and the literature consulted suggest, each of the EI dimensions was considered separately, and all variables (EI dimensions and self-efficacy) were correlated with each other.

**FIGURE 2:**
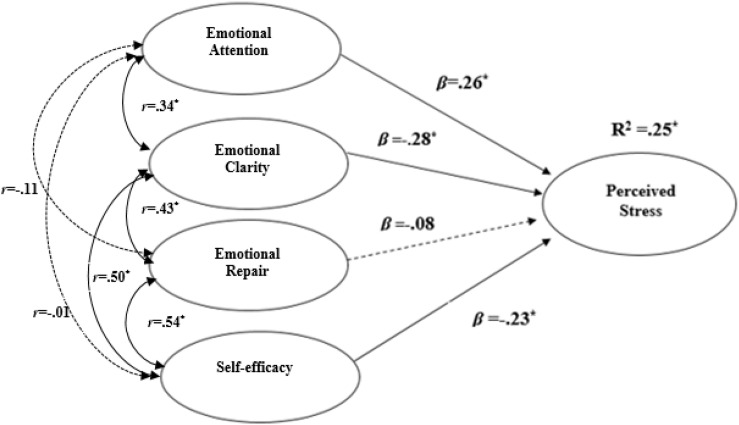
Model of causal relationships between the dimensions of emotional intelligence, overall self-efficacy scale, and perceived stress. *Statistically significant relationship. *p ≤ 0.05; χ^2^ = 1173.50, df = 619, *p* ≤ 0.01; S-Bχ^2^ = 1332.37, df = 619, *p* ≤ 0.01; S-BX^2^/df = 2.15; RMSEA = 0.049 (IC = 0.045–0.053); CFI = 0.90; IFI = 0.90. Discontinuous lines mean that there is no significant relationship between variables.

The results of the causal relationships model showed a good overall fit: χ^2^ = 1173.50, df = 619, *p* ≤ 0.01; S-Bχ^2^ = 1332.374, df = 619, *p* ≤ 0.01; S-BX^2^/df = 2.15; RMSEA = 0.049 (IC = 0.045-0.053); CFI = 0.90; IFI = 0.90. Figure 2 shows the standardized coefficients of each of the relationships that have proven to be statistically significant predictors of the perceived stress variable. The model explained 25% (*R*^2^ = 0.25) of the variance, and it was found that the factors of EA and EC showed a statistically significant relationship in the positive (β = 0.26) and negative (β = −0.28) sense, respectively. Overall, self-efficacy also showed a statistically significant negative relationship (β = −0.23) with perceived stress. The analysis showed that self-efficacy was positively and significantly related to ER (*r* = 0.54; *p* = 0.02) and to EC (*r* = 0.50; *p* = 0.02), but it was not related to EA (*r* = −0.01; *p* ≥ 0.05). Also, EI variables were positively related among them as follows: EA with EC (*r* = 0.34; *p* = 0.02), ER with EC (*r* = 0.43; *p* = 0.04), but not EA with ER (*r* = −0.11; *p* ≥ 0.05).

### Comparative Qualitative Analysis of Fuzzy Sets (fsQCA)

#### Necessary Analysis

Based on the results obtained on the necessary analysis, it appears that there is no necessary condition for the high or low levels of perceived stress, since all consistency values were under 0.90 (Ragin, 2008) (Table 2).

**TABLE 2:** Necessary analysis for perceived stress.

	High levels of		Low levels of	
	perceived stress		perceived stress	
		
	Cons	Cov	Cons	Cov
EA	0.64	0.61	0.52	0.63
∼EA	0.61	0.50	0.68	0.71
EC	0.60	0.59	0.53	0.66
∼EC	0.65	0.52	0.67	0.69
ER	0.60	0.61	0.50	0.65
∼ER	0.65	0.50	0.69	0.69
GSE	0.62	0.58	0.54	0.66
∼GSE	0.64	0.52	0.66	0.69

*∼, absence of condition; Cons, consistency; Cov, coverage; condition needed: consistency ≥0.90; EA, emotional attention; EC, emotional clarity; ER, emotional repair; GSE, general self-efficacy.*

#### Sufficiency Analysis

With regard to the sufficiency analyses, the combination of conditions that led to high and low levels of perceived stress (Table 3) was calculated. Regarding sufficient conditions, to calculate the models, all variables were absent for the high level of stress with the exception of EA, which was present. The frequency cut-off in the truth table was set at 1, and the consistency cut-offs were set at 0.78.

**TABLE 3:** Summary of the main sufficient conditions for the intermediate solution of perceived stress.

*Frequency cutoff: 1*	High levels of perceived stress			Low levels of perceived stress				
		
	*Consistency cutoff: 0.78*			*Consistency cutoff: 0.83*				
		
	1	2		1	2	3	4	
Emotional attention	•	•			∘		∘	
Emotional clarity		∘		∘	•	∘	•	
Emotional repair	•	•		∘	∘	•		
Self-efficacy	∘			•		∘	∘	
Raw coverage	0.29	0.29		0.30	0.28	0.27	0.27	
Unique coverage	0.06	0.05		0.09	0.03	0.05	0.01	
Consistency	0.78	0.76		0.83	0.85	0.81	0.87	
**Overall solution consistency**			**0.76**					**0.79**
**Overall solution coverage**			**0.35**					**0.50**

*∙, presence of condition; ∘, absence of condition; ∼, absence of condition. Expected vector for perceived stress: 0.0.0.1 (0: absent; 1: present). Expected vector for ∼ perceived stress: 0.1.1.1 using the format of Fiss (2011).*

The intermediate solution indicated two combinations of causal conditions that can produce high levels of perceived stress that accounted for 35% of the cases (overall consistency = 0.76; overall coverage = 0.35) and four combinations of causal conditions that lead to low levels of perceived stress that explained 50% of the cases (overall consistency = 0.79; overall coverage = 0.50) (Table 3). As mentioned before, in sufficiency analysis, the concept of raw coverage refers to the variance explained, which means the number of observations that can be explained by a particular combination of conditions. The consistency of the solution expresses the possible reliability, or fit, of a model. According to the previous literature, in fsQCA, a model is informative when its overall consistency is ≥0.75 (Eng and Woodside, 2012). The obtained solution seems, therefore, appropriate.

In the prediction of high levels of perceived stress, the two pathways or combinations that accounted for 35% of the cases were the result of the interaction of low levels of self-efficacy and high levels of ER and EA (raw coverage = 29; consistency = 0.78) that explained 29% of the people with high levels of stress; and the interaction of low levels of EC, high levels of ER, and high levels of EA (raw coverage = 0.29; consistency = 0.76) that explained another 29% of the cases with high levels of stress. In other words, 29% of the people with high levels of stress had low levels of self-efficacy along with high levels of ER and high levels of EA, while another 29% of the people with high levels of stress had low levels of EC as well as high levels of ER and EA. That is, a student who shows low self-efficacy, along with high EA and ER or those with low levels of EC along with high levels of EA and high levels of ER, will show higher levels of perceived stress. On the other hand, to the prediction of low levels of perceived stress, four pathways were observed that explained 50% of the cases with low levels of perceived stress (overall consistency = 0.79; overall coverage = 0.50). The most relevant pathways or combinations to predict this perceived stress were the result of the interaction of low levels of ER and EC and high levels of self-efficacy (raw coverage = 0.30; consistency = 0.83) explaining 30% of the cases with low levels of perceived stress. The other pathway was the combination of low levels of ER and EA and high levels of EC (raw coverage = 0.28; consistency = 0.85) explaining 28% of the cases. The third combination was low levels of self-efficacy and EC, with high levels of ER (raw coverage = 0.27; consistency = 0.81). Finally, the last pathway was the combination of low levels of self-efficacy and EA, with high levels of EC (raw coverage = 0.27; consistency = 0.87).

## Discussion

In the academic context, it is relevant to assess the levels of perceived stress of university students and its repercussion on the students’ levels of performance and psychological well-being. In this fashion, there are numerous studies that analyze strategies to cope with stressful situations in academic environments, all having in common the existence of variables that may modulate the response to the stressor, highlighting the emotional skills of the student (Aguilar et al., 2014; Bryant and Malone, 2015; Hodzic et al., 2016) as well as her belief of self-efficacy (Salanova et al., 2005; León-Rubio et al., 2011).

Responding to the proposed objective of analyzing the existing relationships between the study variables with perceived stress levels through the combination of two complementary methodologies, the results obtained in the SEM go in the line of earlier studies (Extremera et al., 2016; Serrano and Andreu, 2016; Zhang et al., 2016); thus, EC and ER are related in a negative manner with stress levels and EA in a positive manner with the said stress levels. Furthermore, self-efficacy is negatively related to the stress level, supporting findings from previous research (Cabanach et al., 2017). In general, predictive values similar to previous studies have been observed, as regard the amount of explained variance of emotions in relation to stress; in this case, the main variable that explains the levels of perceived stress in university students is EC (Serrano and Andreu, 2016). In this fashion, a greater belief in self-efficacy and emotional competence appears to cushion the level of stress perceived by the students (Aguilar et al., 2014; Zhao et al., 2015; Extremera et al., 2016; Schönfeld et al., 2016). There appears, therefore, to be empirical evidence that supports both H1 and H2.

A primary appraisal, dependent on external factors, and a secondary appraisal, dependent on internal factors, would intervene when evaluating the situation as stressful, according to the model of Lazarus and Folkman (1986). Within the secondary appraisal, an emotional component (EI) and a cognitive component (self-efficacy) are to be found, which influence the level of perceived stress in the academic context. On the one hand, the results showed that high levels of self-efficacy would be related to low levels of perceived stress, in consonance with previous studies (Karademas and Kalantzi-Azizi, 2004; Zhao et al., 2015; Dominguez-Lara, 2018) and thus confirming hypothesis 2. On the other hand, the components of EI should be taken into account separately, since it has been seen that higher levels of EA were positively related to perceived stress, following the line of previous studies (Guerra-Bustamante et al., 2019; Martínez-Marín and Martínez, 2019) and supporting hypothesis 1 (Davis and Nichols, 2016; Villanueva et al., 2017). Otherwise, as indicated by previous studies (Bryant and Malone, 2015; Perera and DiGiacomo, 2015; Schönfeld et al., 2016; Zeidner and Matthews, 2016), EC was positively related to perceived stress, but ER did not do as expected, which partially supports hypothesis 1 (the greater the repair and EC, the lower the level of stress). A person with high expectations of self-efficacy, high scores on ER and clarity, and moderate scores on attention is associated with lower levels of perceived stress. Thus, the student will make a secondary assessment of the situation, and she will have healthy coping strategies that will allow her to reduce her stress levels (Martínez-Marín and Martínez, 2019).

It would be interesting to continue working along these lines in future research due to the difference between the components of EI on stress levels as well as due to the complex behavior of EA indicated by previous studies (Guerra-Bustamante et al., 2019).

In response to the comparison between the two data analysis methodologies, the majority of studies in psychology have focused on analyzing the levels of perceived stress through linear models (Ruiz-Aranda et al., 2014), but they have not studied these aspects through other types of non-linear relationships, much less by the combination of two complementary methodologies such as the SEM and the QCA model. Thus, the combined use of different methodologies enables furthering the relationship between the variables under study. QCA models enable the in-depth analysis of how a series of combined causal conditions (or individually) contribute to a given result. This type of techniques also enables observing the different pathways or combinations that lead to the same result (equifinality). Our results suggest that, even if none of the conditions are necessary for the perceived stress (both high and low levels), there does appear to be two sufficient combinations that would explain 35% of the cases, one of them explaining that 29% is the interaction between high EA, low EC, and high self-efficacy and, conversely, high EA, high ER, and low EC with 29% of explained variance. Incidentally, insofar as the prediction of low stress levels are concerned, the analyses suggest the existence of four pathways, explaining 50% of the cases, the two most explanatory pathways being the result of the interaction between high self-efficacy and low ER and low EC (30% of explained variance) and the other pathway ensuing from the interaction of low EA, low ER, and high EC explaining 28% of the variance. All this seems to go in line of earlier studies, suggesting that the combination of emotional competences and a perception of self-efficacy would lead to more appropriate coping strategies based on planning and problem solving (Augusto-Landa et al., 2011; Fernández-Berrocal et al., 2012), which, in turn, would imply lower levels of perceived stress.

It is observed from a comparison of both methodologies that the fsQCA models have a higher predictive value in enabling the combination of diverse conditions, an increase of between 10% and 25% being observed with respect to the SEM in the prediction of perceived stress levels contingent on whether high or low levels of stress are predicted. Likewise, it is observed that the prediction of high stress levels does not depend on the same factors or conditions as the prediction of low stress levels, something that could not be ascertained from analyses based on linear models. Also, in the SEM, ER is insignificant, while in the QCA models through the combination of different variables, it appears as part of the final result and its presence together with low self-efficacy or high EA may lead to increased stress levels.

Nevertheless, it would appear from a comparison of both methodologies that the QCA models prove to be more illustrative of stress than linear models and that they enable to render account of non-linear relationships; that is, the conditions that may result in high stress levels do not necessarily have to be the same ones that result in low levels of the said stress. Likewise, the QCA models enable the identification of different pathways or combinations that lead to a determined result (equifinality). The QCA models do not focus on the contribution or individual significance of each variable, although the results of the needs analysis may provide a picture in this regard and be a first approximation to determine which variables are more important. This, of course, is not the main objective of this analysis, but it can be used in an exploratory fashion, requiring inherent analysis of the linear models in order to draw conclusions. On the basis of the foregoing, given that the linear and QCA models correspond to different objectives, complementarity must be advocated, rather than focusing on just one or the other. In research, the simultaneous use of both techniques is recommended, as specified in earlier studies (Schneider and Wagemann, 2010; Woodside, 2013; Barton and Beynon, 2015; Felício et al., 2016; Rey-Martí et al., 2016). For all this, the two methodologies would be complementary.

Despite the contributions made by this study, given that the same provides an innovative perspective through the use of two complementary methodologies underused thus far in the discipline and enabling, in turn, the contribution of relevant information as regard perceived stress in university students, this research is not without its limitations. One of the major constraints is related to the study sample, both in terms of sampling procedures, which were not probabilistic, and in terms of geographical location, given that this study was based solely on students of a single university of the Valencian Community, which makes the generalization of the results difficult, although the recourse to the same university and faculty enables controlling much of the peculiar variance. Another constraint is related to the use of questionnaires; although they are a common tool in research, they may introduce social desirability biases. Also, the questionnaires used do not measure stress or academic self-efficacy specifically but stress and self-efficacy in general. The environment with which students usually relate has not been analyzed, and neither has their academic performance (grades). It would be interesting to take it into account in future research in order to provide us with key information on perceptual aspects or intervention strategies. In addition, the analysis of other sociodemographic variables such as age will be taken into account, in case it could influence the results. This should be taken into account in future research.

Future research should focus on using probabilistic sampling procedures and increasing the sample under study in other universities and geographical areas in order to ensure the generalization of results. Similarly, it would be interesting to include objective stress measures such as cortisol levels. Finally, it would be interesting to analyze the moderating role of other variables such as gender or age and compare the said results with the QCA models.

## Conclusion

As both EI and perceived self-efficacy seem to play a determining role in stress levels, and as satisfactory management of stress in academic contexts has a positive impact on the emotional well-being and the academic performance of university students, the present study is of special interest. It combines two analytical methodologies that allow to know in greater depth the phenomenon of the study. Also, the promotion of the design of measures and intervention programs aimed to improve the students’ quality of life. This comparison of innovative methodologies enables to broaden new horizons at the methodological level, which can be applied to different contexts.

## Data Availability Statement

The datasets generated for this study are available on request to the corresponding author.

## Ethics Statement

The studies involving human participants were reviewed and approved by the Catholic University of Valencia (PRUCV/2015/660). Written informed consent for participation was not required for this study in accordance with the national legislation and the institutional requirements.

## Author Contributions

All authors participated and contributed in the study design, the data collection, the statistical analysis, the interpretation of data, and drafted the manuscript. Besides, all authors read and approved the final manuscript.

## Conflict of Interest

The authors declare that the research was conducted in the absence of any commercial or financial relationships that could be construed as a potential conflict of interest.
